# Percutaneous extrapedicular vertebroplasty with expandable intravertebral implant in compression vertebral body fracture in pediatric patient—technical note

**DOI:** 10.1007/s00381-016-3250-8

**Published:** 2016-09-26

**Authors:** Bartosz Polis, Jacek Krawczyk, Lech Polis, Emilia Nowosławska

**Affiliations:** Polish Mother’s Memorial Hospital Research Institute, Rzgowska Street 281/289, 93-338 Łódź, Poland

**Keywords:** Spine trauma, Compressive fracture, Magerl/AO classification, Vertebroplasty, Intravertebral implant, Fracture reduction, Intravertebral cement, Percutaneous procedure, Extrapedicular approach, TLISS/TLICS classification

## Abstract

**Purpose:**

The aim of the article is to present the new extrapedicular percutaneous technique for posttraumatic vertebral column fracture.

**Methods:**

A 15-year-old boy needed a surgical Th8 posttraumatic vertebral body (VB) compressive fracture reduction due to insufficient conservative treatment and consistent severe clinical symptoms. After 6 months of external Jevett long-roll brace stabilization, progressive sagittal balance disturbance of thoracic kyphosis was measured and persistent clinical symptoms were observed. It was decided to present a surgical technique method allowing to attempt to reduce VB fracture, rebalance the vertebral column (VC) without any motion limitation, and decrease clinical symptoms. The procedure was performed percutaneously from extrapedicular approach with intravertebral implant (Spine Jack®—Vexim™) and cement (Interface®—Vexim™) under fluoroscopic imaging (Ziehm™ 8000®).

**Results:**

The whole procedure was uneventful. Now, the child is free from clinical symptoms and the partial reduction of VB fracture was achieved. The patient has been followed for 3 months. In the control CT scans, the VB fracture reduction is stable and no progression of thoracic kyphosis angle is observed. Furthermore since the surgical procedure, the patient is clinical symptom free.

**Conclusion:**

The extrapedicular percutaneus technique of VB fracture reduction with intravertebral fixation allowed to partially reduce the VB compressive fracture, rebalance the VC without any motion limitation, avoid external long-roll brace, and eliminate clinical symptoms. The procedure is minimally invasive, fast, and clinically effective. However, the technique should be restricted only to carefully selected clinical cases.

## Introduction

Posttraumatic VB fractures in pediatric patients are not common. Only 1–2 % of children suffer from VC fractures [1]. The most common is compressive type of VB fracture [2]. Usually, it concerns anterior column fractures (Denis classification) and restoration of anterior support to regain sagittal balance of the VC is generally recommended [3–6]. However, there is still controversial discussion regarding timing and treatment method strategies in such cases [7–10]. Compared to adults, the ratio of VC injuries is estimated as 1:9. In the first 8 years of life, VC injuries concern 85 % cervical part of the spine. Above 8 years of age, the distribution is similar to adults [1]. In USA, over 1500 surgical procedures of posttraumatic VC injuries in pediatric patients are performed every year [1]. The average age of pediatric patient qualified for surgical treatment due to VC injury is 15 years of age [1]. Most of the fractures are referring to sport activities and home games after that vehicle accidents are mentioned [1, 11]. Usually, fracture concerns Th6 and Th12 and the main height reduction of fractured VB is measured at 27 % [11]. In 1-year follow-up, two thirds of the patients have some persistent spine pain and 50 % of them daily [11]. The most frequently used classifications in VC fractures are AO/Magerl (1994) and TLISS/TLICS (2005), relying on Denis three column spine division [3, 12–16]. In AO/Magerl classification scale, it is believed that injuries classified as A1 and A2 (except from pincer fracture—A2.3) are stable ones and do not need surgical treatment—only Jevett long-roll brace [12]. In TLICS (Thoracolumbar Injury Classification System), almost all A1 and A2 AO/Magerl fractures are estimated as 3 or less points and treated as nonoperative [13, 14]. Both systems however do not pay attention to sagittal balance and persistent clinical ailments. In 1986, Bucholtz and Gill pointed sever limitations of Denis classification including absence of considering the dynamic mechanism of spinal injury [17]. Furthermore, most of the surgical techniques are designed for adults. The access routes and implants are designed in the same matter. The size of implant or technique usually makes the procedure impossible to perform in pediatric patients. The most common surgical techniques for compressive fracture in adults are vertebroplasty/kyphoplasty (VP/KP) and posterior transpedicular stabilization (PTS) [18–21]. In case of VP/KP, we have multidirectional vector of acting forces during balloon inflation or cement application, what is adverse reaction—the main vector of force should be directed in vertical axis. Fracture reduction has an impact on the long-term fixation—concept of stable reduction. The average VB high recovery in VP/KP is less than 15 % in fresh (up to 6 weeks) compressive fractures (A1, A2 AO/Magerl). Cement usually is polimethyl methacrylate (PMMA) that cannot also be used in pediatrics. For PTS, it is always a rigid system that in single-level compressive fracture excludes from movement minimum two motor units; moreover, titanium-made implants do not grow or extend during natural human growth. This exposes the patient for additional surgical procedures and limits the movement of VC. All this makes those methods very limited in pediatric patients. The possibility of percutaneous implantation of an intravertebral expansible device that directs vector of acting forces only in vertical axis and use of bone rebuilt cement in combination of preserving full anatomical movement of VC seems to be the optimal solution for selected pediatric patient.

### Case presentation and suggested surgical technique

**Fig. 1:**
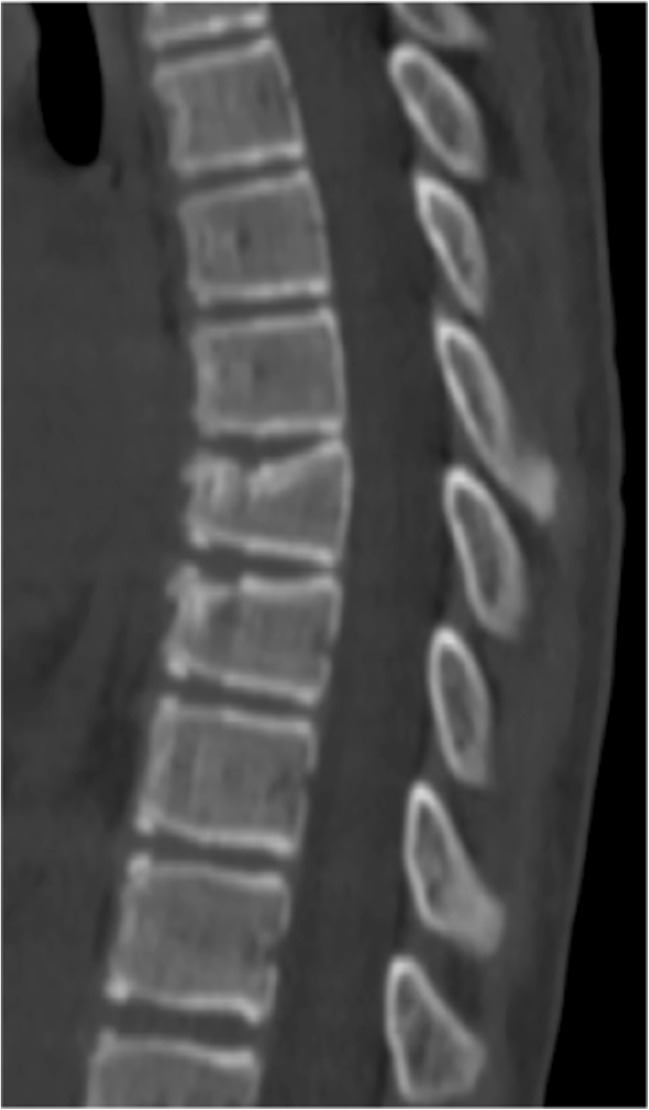
CT scan of fractured VB (sagittal)

**Fig. 2:**
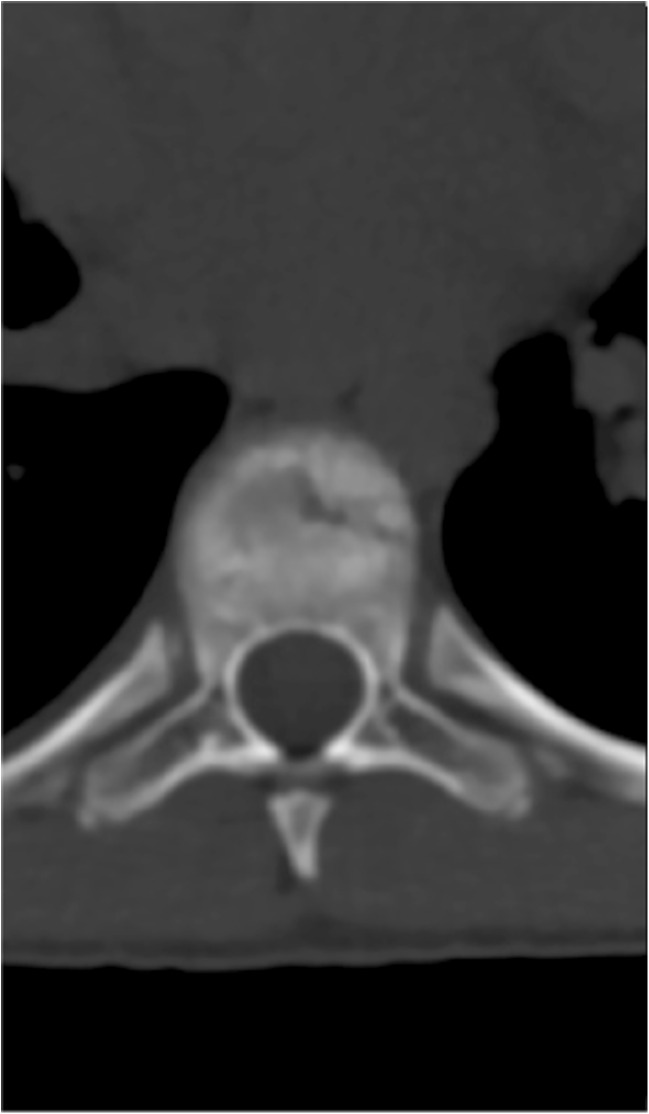
CT scan of fractured VB (axial)

**Fig. 3:**
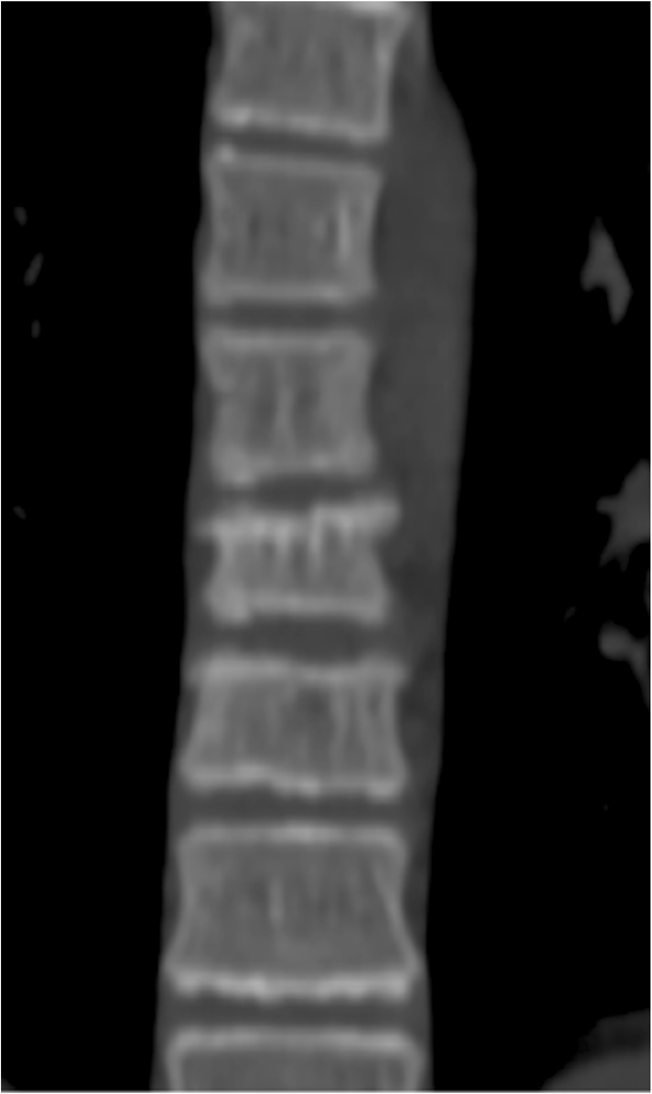
CT scan of fractured VB (coronal)

**Fig. 4:**
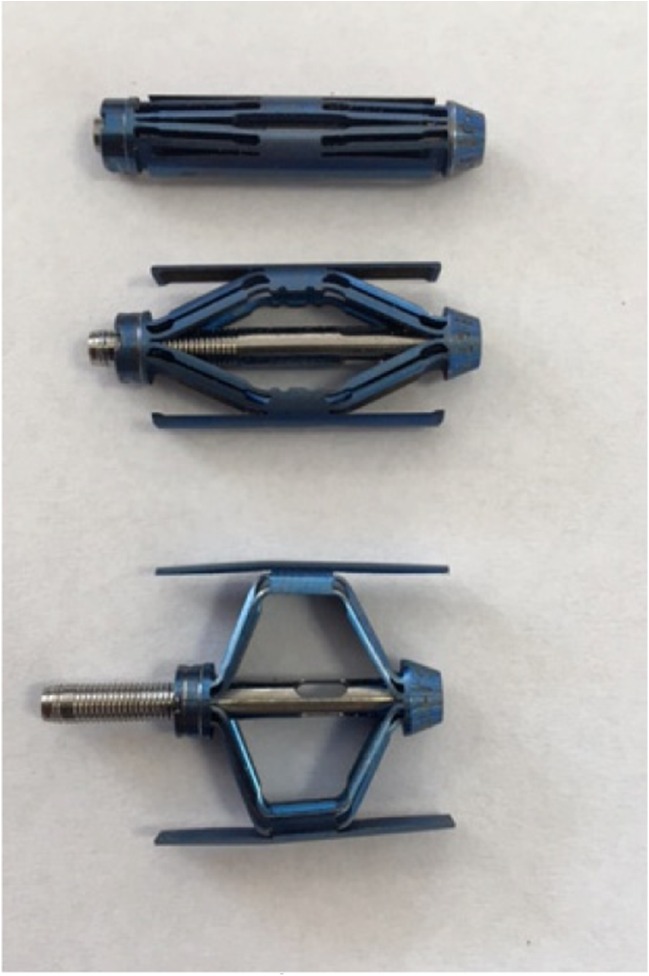
Spine Jack^®^ expandable implant

**Fig. 5:**
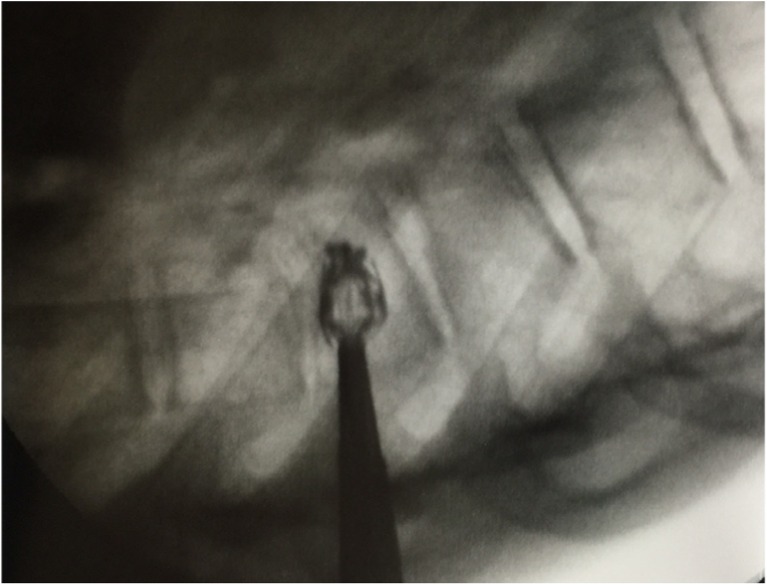
Intraoperative positioning of implant before cementation

**Fig. 6:**
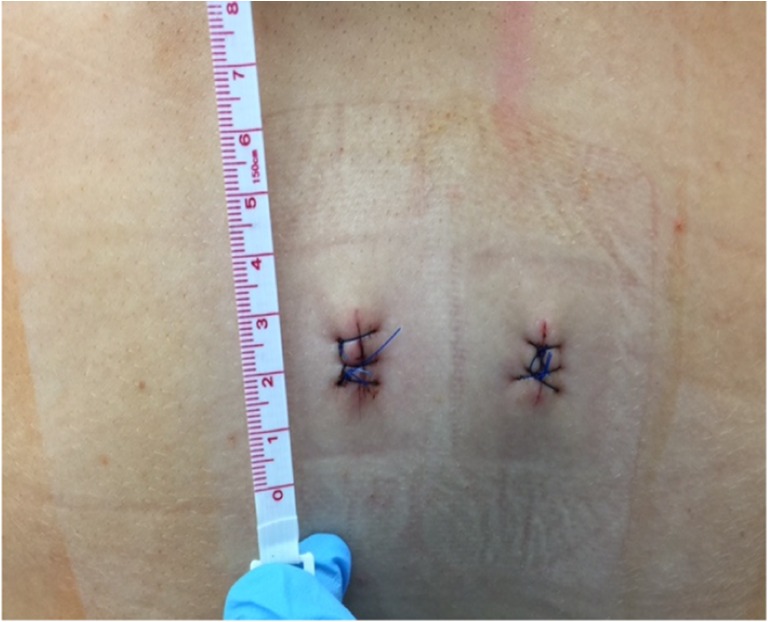
Postoperative wound

**Fig. 7:**
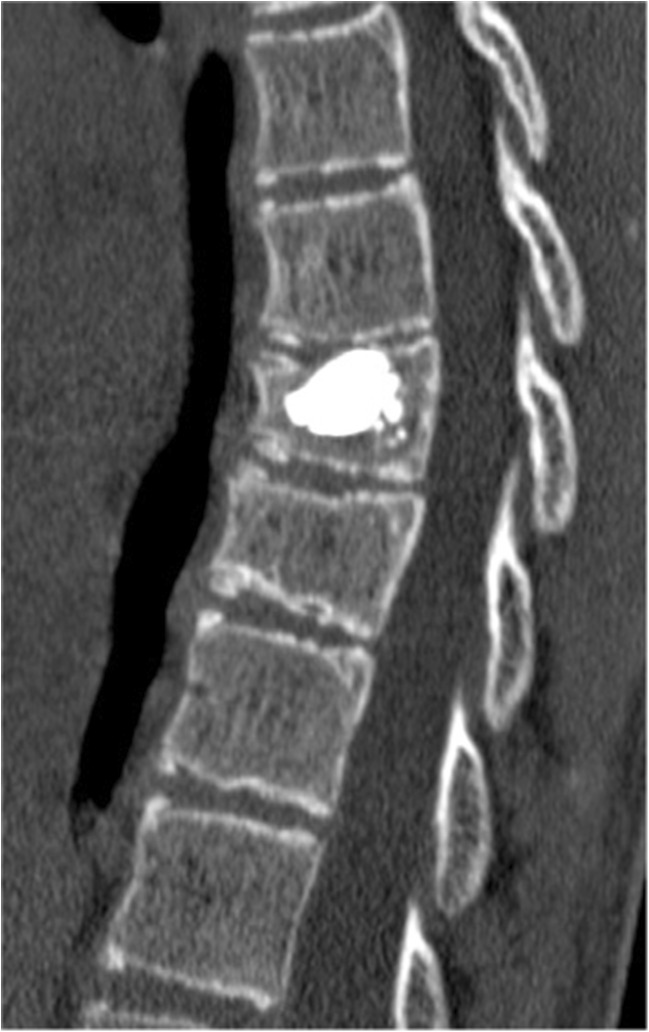
CT control scan of VB (sagittal)

**Fig. 8:**
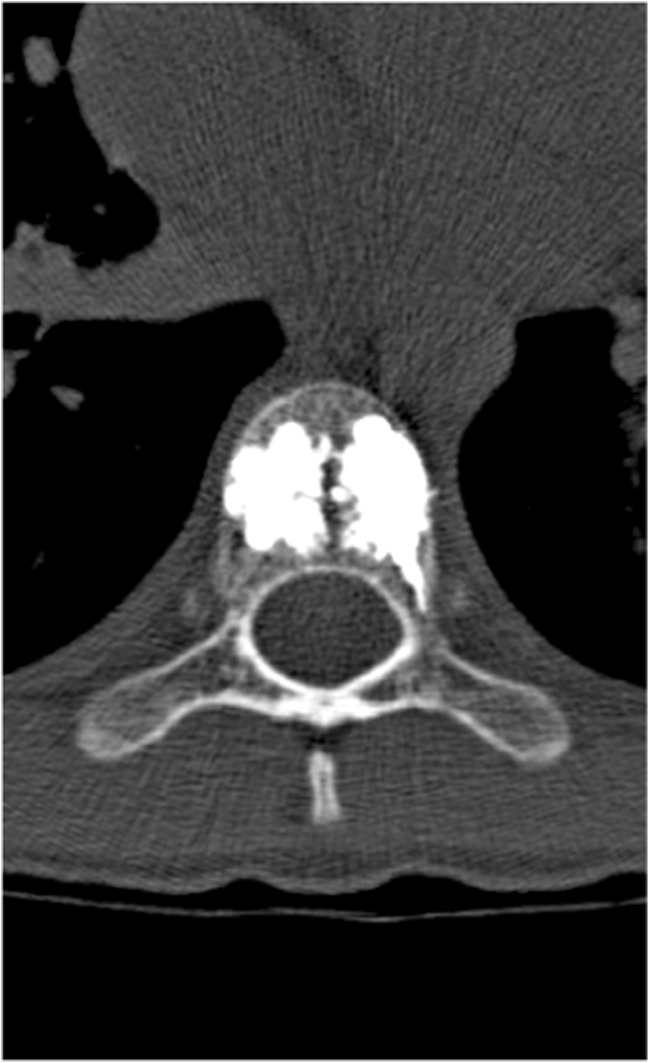
CT control scan of VB (axial)

**Fig. 9:**
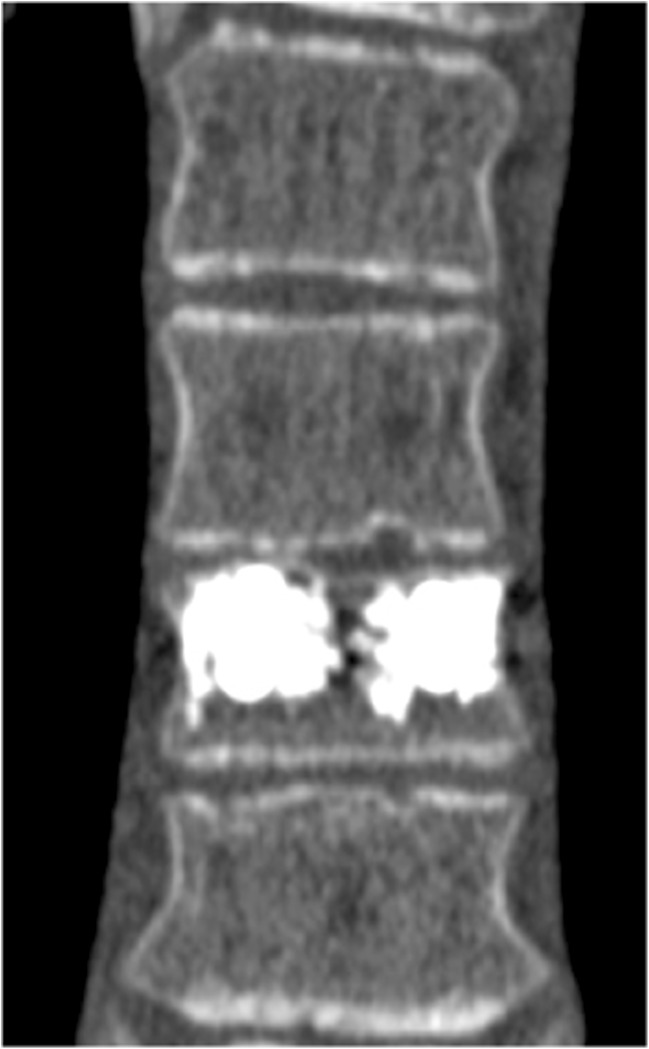
CT control scan of VB (coronal)

**Fig. 10:**
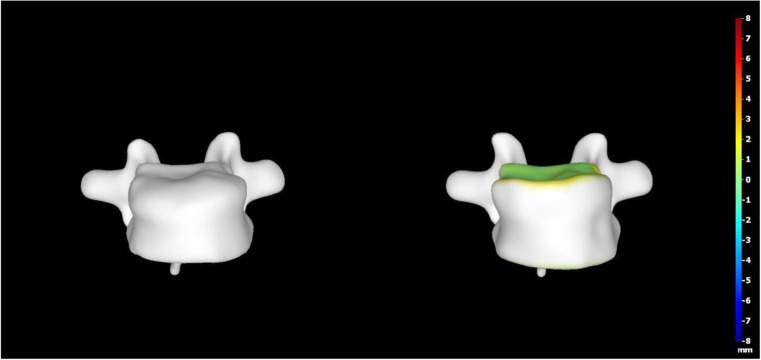
3D mapping reconstruction of pre- and postoperative VB

**Fig. 11:**
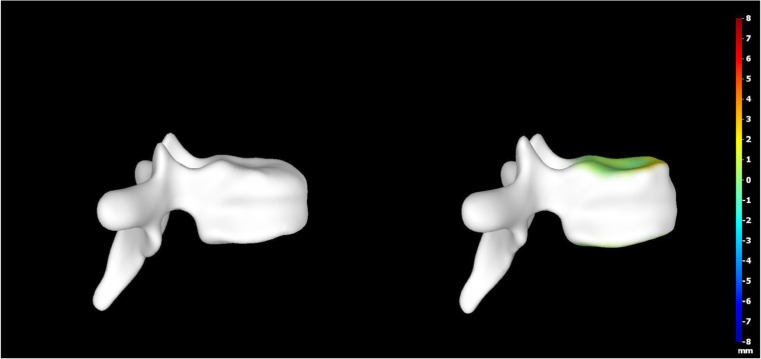
3D mapping reconstruction of pre- and postoperative VB

**Fig. 12:**
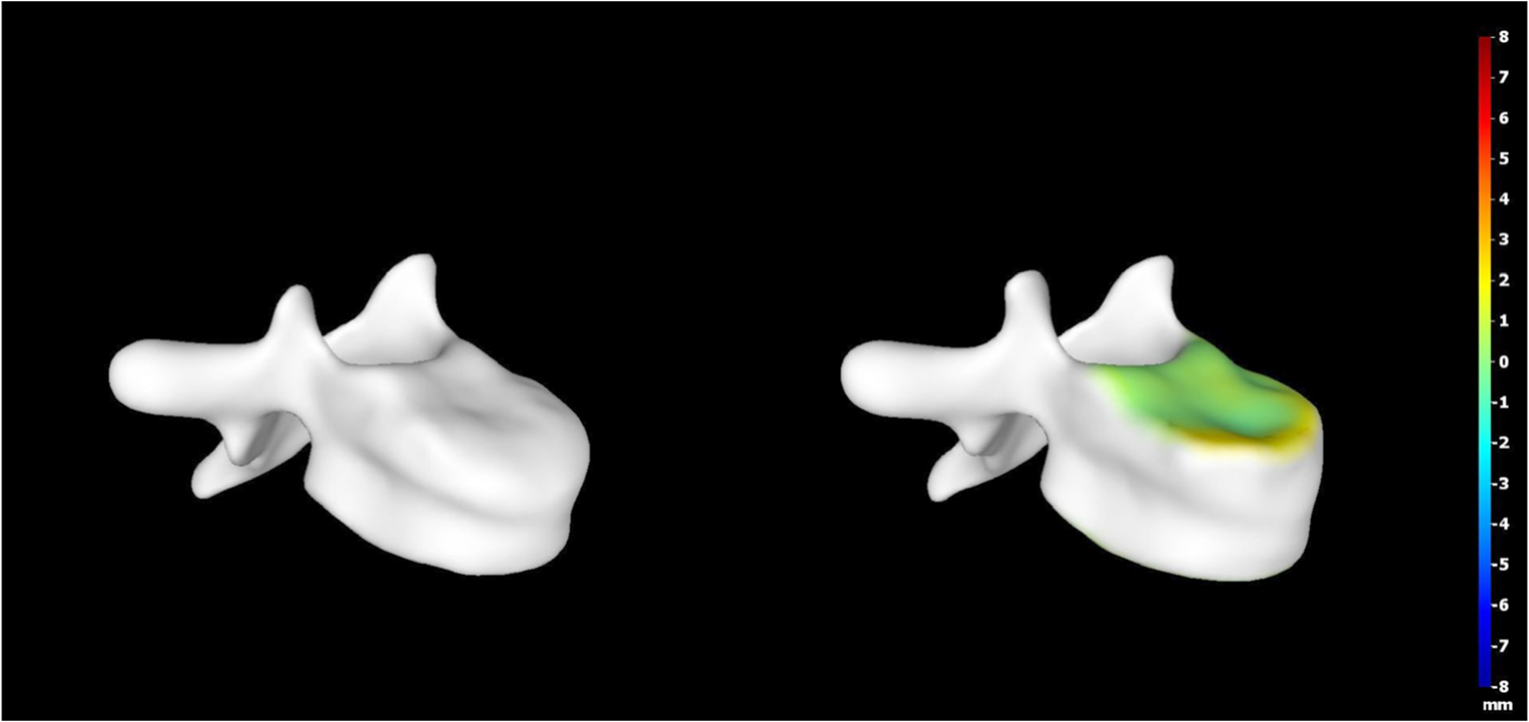
3D mapping reconstruction of pre- and postoperative VB

**Fig. 13:**
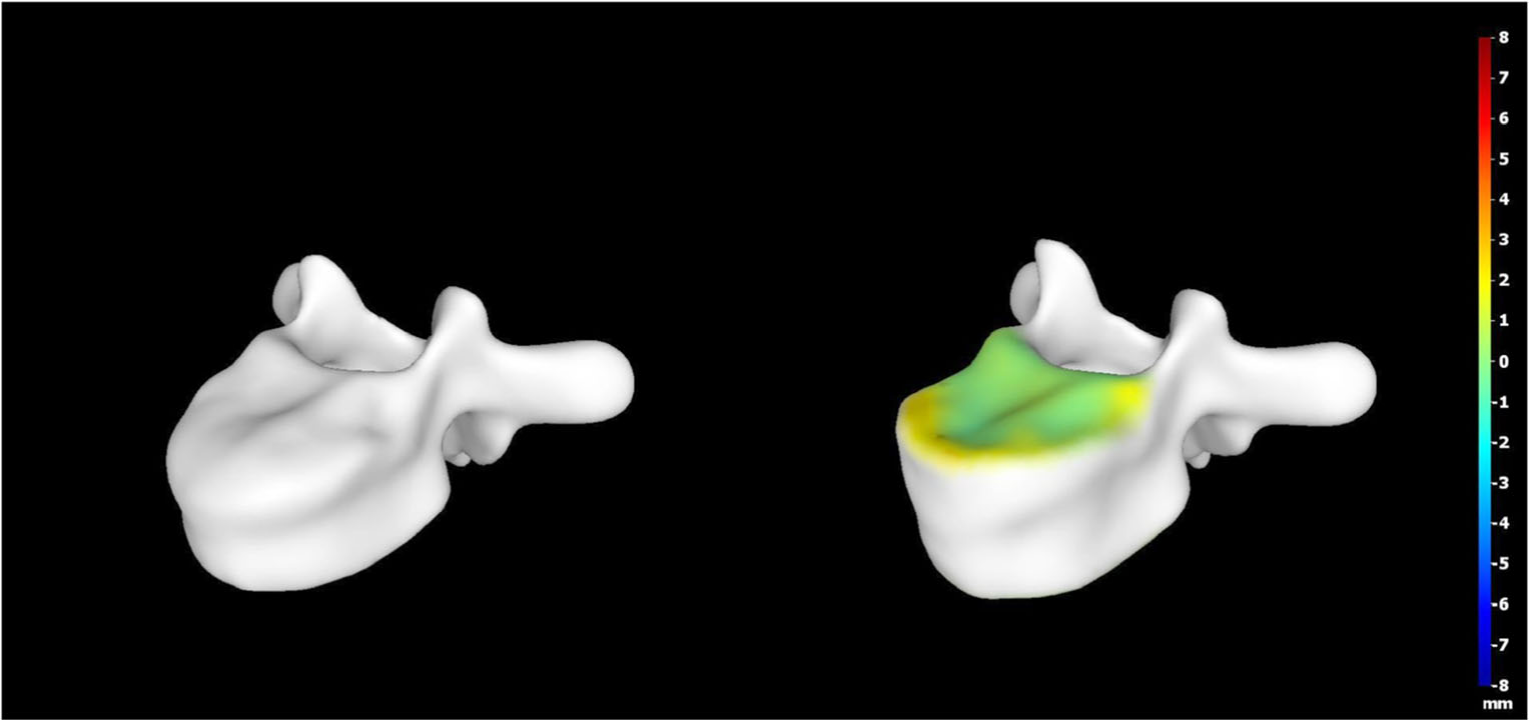
3D mapping reconstruction of pre- and postoperative VB

**Fig. 14:**
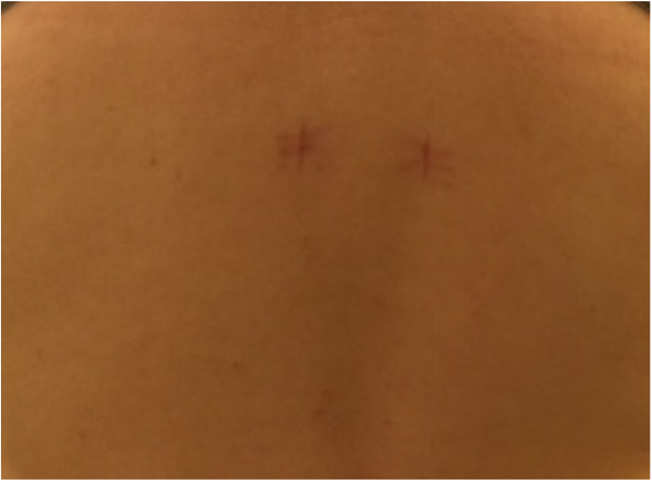
Postoperative wound—control

A 15-year-old boy was admitted to the Polish Mother’s Memorial Hospital Research Institute (PMMHRI) Emergency Unit due to VC injury. The boy was jumping on beaten and in one of the high jumps missed it, landing on hard ground on the back, without loss of consciousness. Because of the injury and strong pain in the thoracic VC, an ambulance was called and the patient was carried on stiff board to the Emergency Unit. At admission, CT scans were made revealing A2.2 (AO/Magerl) fracture of Th8 and A1.2 (AO/Magerl) of Th9 VB. (Figs. 1, 2, and 3) No neurological symptoms were diagnosed. VAS was estimated on 8/10. The angle of thoracic kyphosos (AK) was 24.5° (all angles were measured on digital pictures with Osirix Computer software). According to TLISS/TLICS and AO/Magerl classification, conservative treatment with long-roll Jevett brace and pharmacotherapy was commissioned. The patient after supply was discharged home with recommendations—long-roll brace, pharmacotherapy, and avoidance of physical exercises. After that, he visited several orthopedics who recommended continuation of conservative and pharmacological treatment. After 6 months, the patient was for the first time seen by a neurosurgeon. Still, the patient was in long-roll brace, no neurological symptoms, and VAS was estimated on 7–8/10. Control CT scan was performed. AK was 31.5° which meant 29 % increase compared to first CT. During all that time, the patient was released from any kind of physical exercises. The patient only attended to school classes and was still on pharmacological painkillers. Oswestry Disability Index (ODI) was evaluated as 48 % which meant severe disability. Because of insufficient conservative treatment, surgical procedures were proposed. The surgical procedure was performed under general anesthesia in standard position for posterior VC approach. To eliminate all inconvenience associated with standard techniques, it was decided to use intravertebral Spine Jack® fixation system offered by Vexim™. To avoid use of pure PMMA cement (Cohesion™), we decided to use Interface™ bone rebuilt cement also offered by Vexim™ in order to target patients with high reossification potential, like young ones. The system was designed for adults and standard implant dimensions are customized to fully growth pedicles. The smallest implant is 4.2 mm in diameter. The pedicle of fractured VB was 4.1 mm. The implant was bigger in diameter than the pedicle itself, not mentioning maintenance of safe 2-mm bone margin around the planned approach. The extrapedicular approach was chosen as the method to place the implant in the planned final location. Spine Jack® 4.2-mm implant was chosen (Fig. 4) and Interface™ cement. The whole procedure took 45 min. The implants were placed under fluoroscopic imaging (Ziehm™ 8000®). After proper positioning of the implant in Th8 VB (Fig. 5), it was fully expanded followed by cementation of the implant and VB with Interface™ bone rebuilt cement (total amount of 1.8cm^3^ on side—total volume of 3.6cm^3^). Cement was applied with very low pressure (indispensable to push through the cement application tubes). After obtaining full hemostasis, sutures were applied on the fascia and skin. The skin incision was 1.5 cm in length on side (Fig. 6). After procedure, CT scan was performed revealing proper implant position and proper cement placement (Figs. 7, 8, and 9). AK was measured at the level of 27.1°—14 % of rebalance was achieved. In postoperative 3D mapping, reduction of fracture is clearly visible especially regarding anterior column of VB (Denis classification) and almost ideal distribution of forces acting on endplates (Figs. 10, 11, 12, and 13). Surgical procedure was performed in the 25th week after injury. It was an old fracture; in fractures operated till the 6th week after injury, the percentage of height recovery is significantly higher. Complete mobilization of the patient was done 4 h after the procedure. There were no neurological symptoms. Local wound pain was evaluated as VAS = 1/10. There was no need for the brace or pharmacotherapy. The patient was discharged from the hospital 72 h after admission. In the 10th day after the procedure, stitches were removed. The patient was on control visit 1 and 3 and 6 months after the procedure. Control CT was performed. AK is stable at the level of 27.2° on all control studies. There were no pain (VAS = 0/10) and no brace. Postoperative wound was hardly visible (Fig. 14). ODI was evaluated as 0 % which meant complete recovery. The patient returned to all activities of daily life and to active outdoor sports.

## Discussion

**Fig. 15:**
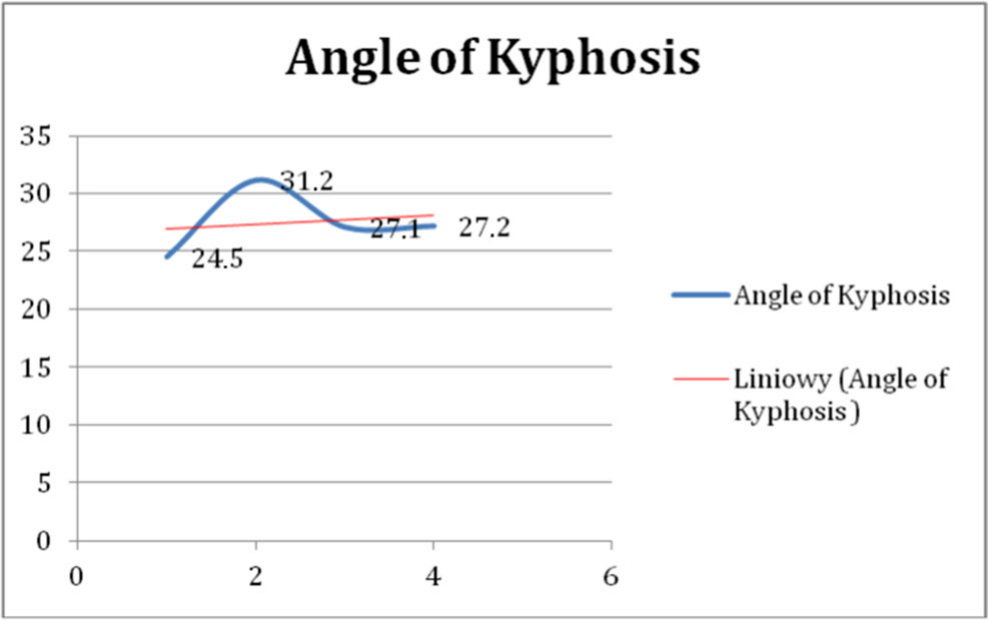
Angle of kyphosis—at time of injury (24.5°), 6 months later (31.2°), after surgical treatment (27.1°), and 3 months follow-up (27.2°)

Extrapedicular approach to fractured VB with expandable implant is a safe and fast surgical treatment method. It should be restricted only to carefully selected clinical cases. It should not be the method of first choice. Only less than 16 % of patients with A1 and A2 AO/Magerl classification fractures develop progressive sagittal balance disorder and permanent clinical symptoms [22]. Directly after injury, conservative treatment should be applied—long-roll Jevett brace and pharmacotherapy. After 1 month, control study of VC should be performed. In cases of insufficient conservative treatment, surgical approach should be considered [23]. The best results of VB fracture reduction is achieved when the surgical procedure is performed in first the 6 weeks after injury; therefore, 71 % of the surgeons operate patients within the first 2 weeks after injury [5]. Furthermore, surgical treatment should be considered when directly after injury, 20 % or more in VB height reduction or progressive VC imbalance is diagnosed. Fracture reduction has an impact on long-term fixation—concept of stable reduction. In pediatric patient’s over 12 years of age, the spine does not reach final growth; therefore despite quite good ossification (corresponding to fully developed one), it cannot be treated as adult one. Ideal cement positioning is only academic. In real life, flow is difficult to manage; therefore, we should manage the quantity. In traumatic fractures, we should apply 12 % of VB volume in simple fractures (A1.2–3 AO/Magerl) and 20 % or more in complex ones (A2, A3 AO/Magerl). In cement injection technique, we should go slow, with almost no pressure and one side at time. After the procedure, we also gain better distribution of biomechanical forces acting on VB endplate. In our case despite dealing with old fracture, we managed to improve sagittal balance of VC, reduce VB fracture, and keep this condition in follow-up controls (Fig. 15). Pain as a clinical symptom was completely removed, and the patient returned to all activities of daily life and to active outdoor sports. The percutaneous technique of VB fracture reduction with intravertebral fixation allowed to partially reduce the VB compressive fracture, rebalance the VC without any motion limitation, avoid external brace, and eliminate clinical symptoms. The procedure is minimally invasive, fast, and clinically effective. However, the technique should be restricted only to carefully selected clinical cases.
